# Natural moisturizing factor as a biomarker for filaggrin mutation status in a multi‐ethnic paediatric atopic dermatitis cohort

**DOI:** 10.1111/cea.14001

**Published:** 2021-08-28

**Authors:** Minke M. F. van Mierlo, Peter J. Caspers, Marieke S. Jansen, Gerwin J. Puppels, Anouk E. M. Nouwen, Madelon B. Bronner, Luba M. Pardo, Michel van Geel, Suzanne G. M. A. Pasmans

**Affiliations:** ^1^ Department of Dermatology‐Center of Pediatric Dermatology Erasmus MC University Medical Center Rotterdam‐ Sophia Children's Hospital Rotterdam The Netherlands; ^2^ Department of Dermatology Center for Optical Diagnostics and Therapy Erasmus MC University Medical Center Rotterdam The Netherlands; ^3^ RiverD International B.V Rotterdam The Netherlands; ^4^ Department of Dermatology Maastricht University Medical Centre Maastricht The Netherlands; ^5^ Department of Clinical Genetics Maastricht University Medical Centre Maastricht The Netherlands

**Key message**

- Raman spectroscopy is a rapid and non‐invasive technique to measure natural moisturizing factor content in AD
- Natural moisturizing factor can predict *FLG* genotype with a sensitivity of 97% and specificity of 87%
- The thenar eminence's nonlesional skin is a suitable location to predict *FLG* genotype

To the Editor,

Atopic dermatitis (AD) is a common inflammatory skin disease among children with increasing prevalence in the past decades. The strongest and most widely replicated genetic risk factor for AD is a null mutation in the filaggrin gene (*FLG*) located on chromosome 1q21. *FLG* encodes the protein filaggrin, which is involved in the formation and homeostasis of the skin barrier.^1^ Previous research showed that AD patients with a mutation in *FLG* have a different phenotype, characterized by early onset disease with persistence into adulthood, increased severity and increased risk of asthma and allergic sensitization.^1^ Additionally, it has been suggested that patients with a mutation in *FLG* respond differently to immunosuppressive treatment compared to wild‐type patients.^1^ This suggests that *FLG* mutation profiling could be used to stratify patients in terms of clinical course and to develop personalized treatment strategies. However, genotyping is time consuming and expensive, and DNA collection poses ethical considerations, which hampers its use in daily practice. Recent literature suggests that decreased concentrations of filaggrin‐derived components of the natural moisturizing factor (NMF) are a proxy for the presence of *FLG*‐null mutations.^2^, ^3^ During the terminal differentiation of keratinocytes, profilaggrin is dephosphorylated and enzymatically degraded into a highly hygroscopic mixture of amino acids and amino acid derivatives, including pyrrolidone carboxylic acid (PCA), histidine and its metabolite urocanic acid (UCA).^2^ The free amino acids and their derivatives constitute the majority of NMF in the stratum corneum (SC).^2^ A previous study in a selected Irish paediatric population of AD patients showed that NMF could discriminate between *FLG* mutation carriers and wild‐type with a sensitivity of 98.73% and a specificity of 86.89% using 1.07 arbitrary units (a.u.) as the cut‐off value.^4^ It has not been investigated whether this cut‐off value could be applied to assess *FLG* mutation status in different clinical cohorts.

The aim of our study was to screen the entire encoding region of *FLG* for potential mutations and to validate whether NMF could be used as a biomarker for *FLG* genotype in an unselected multi‐ethnic clinical cohort of children with mild‐to‐severe disease. We conducted a cross‐sectional study at the tertiary referral centre for Pediatric Dermatology in the Erasmus University Medical Center (Erasmus MC)‐Rotterdam, the Netherlands. Study procedures were approved by the Medical Ethical Committee of the Erasmus MC (MEC‐2017‐370). All patients and/or their parents (guardians) signed informed consent.

Children (0–18 years) diagnosed with AD according to the UK Working Party criteria consulting the dermatologist between June 2018 and September 2019 were eligible to participate in this study. *FLG* mutations were determined on DNA isolated from buccal swabs using Isohelix SK‐1S swabs (Cell Projects Ltd). Single‐molecule molecular inversion probes (smMIPS) and barcoded next‐generation sequencing (NGS) were performed to screen the entire encoding region of *FLG* for all mutations resulting in premature protein termination as previously described.^5^ All patients with a mutation in *FLG* were referred to as patients with ≥1 mutation(s) (*FLG* ^−^). Wild‐type patients were referred to as *FLG* ^+^. The NMF content was measured in the SC of the thenar eminence (nonlesional skin) using confocal Raman spectroscopy (gen2‐ SCA Skin Composition Analyzer; RiverD International B.V.) as described previously.^4^ All investigators were blinded for *FLG* genotype until the end of the study. Acute AD severity was assessed using the Eczema Area and Severity Index (EASI) score on the same day as the NMF measurements. A receiver operating characteristic (ROC) curve was constructed to measure the diagnostic ability of NMF to predict *FLG* mutation status (wild‐type versus ≥1 mutation) by mapping the sensitivity versus 1‐specificity for all possible values of the cut‐off point. The optimal cut‐off point was determined by maximizing the Youden function. A multivariate linear regression model was used to examine the association between EASI (as independent variable) and NMF content, corrected for *FLG* mutation status, age and sex.

A total of 101 patients were included in this study (Figure S1). We identified 12 different *FLG* mutations, corresponding to 30 (30%) patients with ≥1 mutation in FLG (Table S1). This included 25 heterozygous mutation carriers and 5 patients with more than one mutation in *FLG*. There were no differences in age, sex, ethnicity and disease severity between both groups (Table S2). *FLG* ^+^ patients had a median NMF of 1.26 a.u. (IQR 1.18–1.37), compared with a median NMF level of 0.82 a.u. (IQR 0.56–0.94) in *FLG* ^−^ patients (Figure 1, *p* < .01). Furthermore, the NMF content in patients with a single mutation in *FLG* was significantly different compared to both wild‐type patients and patients with 2 mutations in *FLG* (*p* < .01, Figure S2). ROC curves were constructed to test the diagnostic ability of NMF measured using Raman spectroscopy. The optimal cut‐off value for NMF to distinguish between *FLG* ^−^ and *FLG* ^+^ patients was 1.03 a.u. with an area under the curve (AUC) of 0.93 (95% CI 0.87–0.99) (Figure 2). This resulted in a sensitivity of 97%, specificity of 87%, positive predictive value of 76% and negative predictive value of 98% (Table S3). We did not find a significant association between the EASI score and NMF measured at the thenar eminence (corrected for FLG mutation status, age and sex) (beta −0.555 (95% C.I. −0.005–0.003), *p* = .58, Table S4).

**FIGURE 1:**
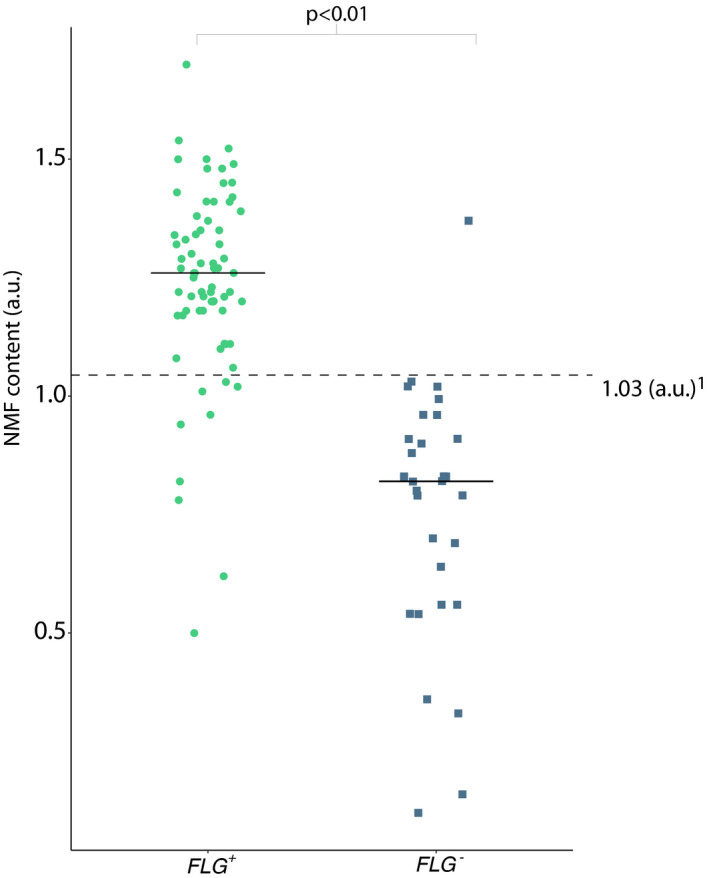
Natural moisturizing factor (NMF) content by *FLG* genotype. Wild‐type patients (*FLG* ^+^) had median NMF content of 1.26 a.u. (IQR 1.18–1.37), compared with 0.82 a.u. (IQR 0.56–0.94) in *FLG* ^−^ (*p* < .01, using a Mann‐Whitney *U* test). ^a^ Cut‐off value of 1.03 a.u

**FIGURE 2:**
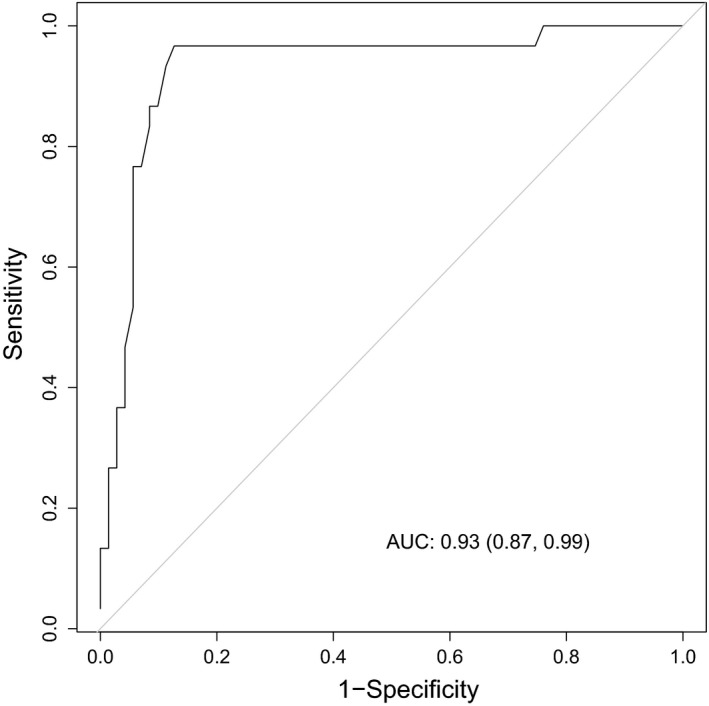
Receiver operating characteristic (ROC) curve determining the discriminatory power for natural moisturizing factor (NMF) content per genotype. Cut‐off value of 1.03 a.u. was determined by maximizing the sum of sensitivity and specificity with an AUC of 0.93 (95% CI 0.87–0.99). This resulted in a sensitivity of 96.67, specificity of 87.32, positive predictive value of 76.32 and negative predictive value of 98.41

The use of the cut‐off value of 1.03 a.u. resulted in high sensitivity and specificity and was very close to the previously determined cut‐off value of 1.07 a.u.^4^ Interestingly, 13% of the wild‐type patients had low NMF content in the SC, consistent with previous literature.^4^ Since we used the smMIP‐NGS technique, this could not be attributed to a rare or new mutation in *FLG*. Our results underline the importance of other factors affecting the NMF content in the SC, which are not a direct result of a *FLG* mutations. Previous research showed a positive correlation between intragenetic copy number variation (CNV) and the amount of filaggrin breakdown products.^1^ In addition, there is an important role for proteases in this multistage breakdown process (e.g. bleomycin hydrolase (BH) and caspase‐14), which can be less effective due to variants influencing enzyme activity or the effect of external humidity.^1^ Furthermore, the presence of other mutations in the epidermal differentiation complex (EDC) could account for low NMF values and should be investigated further. Since Raman spectroscopy has the potential to measure the functional consequences of all mechanisms and mutations leading reduced NMF, this could be more useful in daily practice than genetic analysis alone.

Our results showed no correlation between EASI score and NMF value, making the nonlesional skin of the thenar eminence a suitable location to predict *FLG* mutation status without direct interference of acute disease severity. Previous studies have shown that filaggrin degradation products in the SC of the nonlesional skin of the forearm are affected by both a mutation in *FLG* and disease severity.^6^ The effect of disease severity can be attributed to the activated immune system in both lesional and nonlesional skin. Previous research, using a tape stripping technique, showed an up‐regulation of markers for AD severity including T helper 2 (Th2)‐skewed markers (interleukin [IL]‐13, CCL17, CCL22, IL‐5) in the nonlesional skin of the forearm, which was associated with a reduced NMF content.^6^ The current results did not show a correlation between acute disease severity and NMF measurement on the thenar eminence. This might be explained by the thicker SC with a slower turnover time as compared to the SC on the forearm, making the thenar eminence less susceptible to an acute up‐regulation of the immune response. Determining both cytokines and NMF values in the SC of thenar eminence and correlating this to disease severity could support our hypothesis.

Major strengths of this study are the application of the non‐invasive clinically compatible NMF measurement in an unselected multi‐ethnic patient cohort and the use of a novel technique to detect all mutations leading to a premature protein termination in *FLG*. Especially in this multi‐ethnic population, screening of only the most common mutations is not sufficient and will lead to an underrepresentation of *FLG* mutations. A limitation to mention is that patients were instructed not to apply any topical therapies on the thenar eminence 24 h before the measurement, but information on the use of these ointments during the rest of the week prior to the visit was missing. Previous research has suggested that topical steroid treatment could decrease the level of NMF in the SC of both mice and humans.^7^ Future research should focus on other factors, next to *FLG*‐null variants, leading to a reduction in the NMF. Furthermore, it is of interest to validate the cut‐off value of 1.03 a.u. in other clinical cohorts and to evaluate its use as a predictor for treatment outcomes to enable personalized treatment.

In conclusion, we validated that the NMF content in the SC of the thenar eminence can be used as biomarker for *FLG* status in an unselected clinical cohort of children with AD from different ethnical backgrounds. This will be useful to identify patients with high risk for a more severe phenotype and to stratify patients in future clinical studies to evaluate treatment response.

## CONFLICT OF INTEREST

M.M.F.v.M., M.S.J., A.E.M.N., M.B.B., L.M.P., M.V.G. S.G.M.A.P. have no conflict of interest to declare. P.J.C. and G.J. P.: The gen2‐SCA device is manufactured by RiverD International B.V.

## AUTHOR CONTRIBUTIONS

MMFvM, PJC, LMP and SGMAP designed the study. MMFvM, MSJ and AEMN were responsible for the collection of data. MvG supervised the filaggrin mutation analysis. MMFvM performed the data analysis and wrote the manuscript. SGMAP supervised the study. All authors critically commented on the manuscript.

## ETHICAL APPROVAL

Study procedures were reviewed and approved by the Medical Ethics Committee of the Erasmus MC University Medical Center Rotterdam, the Netherlands (MEC‐2017‐370). Signed informed consent was obtained from all participants.

## Supporting information

Supplementary Material

## Data Availability

The datasets used and/or analysed during the current study are available from the corresponding author on reasonable request.
