# Supplementary material for: Natural moisturizing factor as a biomarker for filaggrin mutation status in a multi‐ethnic paediatric atopic dermatitis cohort
Source: Clin Exp Allergy. 2021 Aug 28;51(11):1510–3. doi: 10.1111/cea.14001 (PMC9291917; doi:10.1111/cea.14001)
Supplement: Supplementary file 1 — Supplementary Material [file CEA-51-1510-s001.docx]

**SUPPLEMENTARY FILE**

**Table S1. *FLG* loss of function mutations found in our study population**

|  |  |  | | | | | |
| --- | --- | --- | --- | --- | --- | --- | --- |
| **ID** | **Ethnic origin** | **Mutation 1** | **Protein change** | **rpt** | **Mutation 2** | **Protein change** | **rpt** |
| 391 | Unknown | c.6453del | p.(Ser2152Profs*7) | 6 |  |  |  |
| 615 | Dutch | c.2282_2285del | p.(Ser761Cysfs*36) | 1 |  |  |  |
| 652 | Dutch | c.3418C>T | p.(Arg1140*) | 3 |  |  |  |
| 662 | Dutch | c.1501C>T | p.(Arg501*) | 1 | c.2282_2285del | p.(Ser761Cysfs*36) | 1 |
| 664 | Dutch | c.1501C>T | p.(Arg501*) | 1 |  |  |  |
| 665 | Dutch | c.2282_2285del | p.(Ser761Cysfs*36) | 1 |  |  |  |
| 705 | Dutch | c.1501C>T | p.(Arg501*) | 1 |  |  |  |
| 714 | Other non-Western | c.2282_2285del | p.(Ser761Cysfs*36) | 1 | c.2976_2977del | p.(Arg992Serfs*31) | 2 |
| 719 | Surinamese-Creole | c.1501C>T | p.(Arg501*) | 1 |  |  |  |
| 723 | Dutch | c.1501C>T | p.(Arg501*) | 1 |  |  |  |
| 729 | Dutch | c.1501C>T | p.(Arg501*) | 1 |  |  |  |
| 733 | Surinamese-Creole | c.6109C>T | p.(Arg2037*) | 5 |  |  |  |
| 736 | Dutch | c.2282_2285del | p.(Ser761Cysfs*36) | 1 |  |  |  |
| 737 | Dutch | c.1501C>T | p.(Arg501*) | 1 | c.1501C>T | p.(Arg501*) | 1 |
| 745 | Other non-Western | c.1217C>G | p.(Ser406*) | 0 |  |  |  |
| 749 | Other non-Western | c.2282_2285del | p.(Ser761Cysfs*36) | 1 |  |  |  |
| 752 | Dutch | c.2282_2285del | p.(Ser761Cysfs*36) | 1 |  |  |  |
| 755 | Surinamese-Creole | c.6950_6957del | p.(Ser2317*) | 6 |  |  |  |
| 756 | Turkish | c.1501C>T | p.(Arg501*) | 1 |  |  |  |
| 764 | Dutch | c.2282_2285del | p.(Ser761Cysfs*36) | 1 |  |  |  |
| 766 | Dutch | c.6950_6957del | p.(Ser2317*) | 6 | c.9894del | p.(Gly3299Glufs*92) | 9 |
| 771 | Dutch | c.2282_2285del | p.(Ser761Cysfs*36) | 1 |  |  |  |
| 775 | Dutch | c.2282_2285del | p.(Ser761Cysfs*36) | 1 |  |  |  |
| 777 | Surinamese-Creole | c.10354C>T | p.(Gln3452*) | 10^1^ |  |  |  |
| 780 | Cape Verdean | c.1501C>T | p.(Arg501*) | 1 | c.2282_2285del | p.(Ser761Cysfs*36) | 1 |
| 781 | Dutch | c.1501C>T | p.(Arg501*) | 1 |  |  |  |
| 792 | Dutch | c.2282_2285del | p.(Ser761Cysfs*36) | 1 |  |  |  |
| 799 | Surinamese-Creole | c.8702C>G | p.(Ser2901*) | 8^1^ |  |  |  |
| 802 | Dutch | c.1501C>T | p.(Arg501*) | 1 |  |  |  |
| 805 | Dutch | c.2282_2285del | p.(Ser761Cysfs*36) | 1 |  |  |  |
| Underlined mutations have not been reported before | | | | | | | |

**Table S2. Patient characteristics**

|  | *FLG^+^* (n=71) | *FLG^-^* (n=30) | p-value |
| --- | --- | --- | --- |
| Gender, n(%)  Male | 35 (49.3) | 11 (36.7) | 0.24^1^ |
| Age, mean±SD (years) | 8.6±5.3 | 8.2±4.4 | 0.55^2^ |
| Ethnic origin^4^  Cape Verdean  Dutch  Dutch Antillean  Moroccan  Surinamese-Creole  Turkish  Other non-Western  Western | 2 (2.8)  30 (42.3)  6 (8.4)  6 (8.4)  7 (9.9)  1 (1.4)  11 (15.5)  4 (5.6) | 1 (3.3)  19 (63.3)  -  -  5 (16.7)  1 (3.3)  3 (10.0)  - | 0.17^1^ |
| Number of mutations, n(%)  1  2 | -  - | 25 (83.3)  5 (16.7)^5^ |  |
| NMF content a.u., median IQR | 1.26 (IQR 1.18-1.37) | 0.82 (IQR 0.56-0.92) | **<0.01**^3^ |
| EASI score, median IQR^6,7^ | 6.1 (IQR 2.3-14.5) | 7.8 (3.3-15.6) | 0.22^3^ |
| Abbreviations: a.u.= arbitrary unit; EASI= Eczema Area and Severity Index, *FLG^+^* = wild-type patients, *FLG^-^* = patients with ≥1 mutation(s) in filaggrin gene; IQR=interquartile range, NMF=natural moisturizing factor, n=number, SD=standard deviation.  ^1^Chi-square test; ^2^independent t-test; ^3^Mann-Withney U test  ^4^Information on ethnicity was missing for 4 (5.6%) patients in *FLG*^+^ and 1 (3.3%) patient in *FLG*^-^ group  ^5^including one homozygous mutation carrier  ^6^EASI score was missing for 4 (5.6%) patients in *FLG*^+^ group and 3 (10.0%) patient in *FLG*^-^ group  ^7^The distribution of EASI score is shown in Table S2 | | | |

**Table S3. Filaggrin genotype per NMF category based on the cut-off value**

|  | *FLG^+^* (n=71) | *FLG^-^* (n=30) |
| --- | --- | --- |
| Low NMF content a.u.^1^ (n=38) | 9 | 29 |
| Normal NMF content a.u.^1^ (n=63) | 62 | 1 |
| Abbreviations: a.u.= arbitrary unit; *FLG*^+^= wild-type patients, *FLG^-^*= patients with ≥1 mutation(s) in filaggrin gene*,* NMF= natural moisturizing factor.  ^1^Based on the cut-off value of 1.03 a.u. | | |

**Table S4.** **multivariate linear regression model testing the association between EASI score and NMF value.**

|  | Standardized beta (95% CI) | p-value |
| --- | --- | --- |
| *FLG* mutation status^1^ | -0.70 (-0.59, -0.38) | <0.00 |
| Sex | -0.06 (-0.13, 0.01) | 0.45 |
| Age | -0.06 (-0.13, 0.01) | 0.43 |
| EASI score^2^ | -0.04 (-0.01, 0.00) | 0.58 |
| ^1^*FLG* mutation status (*FLG*^+^ or *FLG*^-^)  ^2^EASI score (range 0-72) | | |

**Figure S1.** Patients included in analysis

Abbreviations: NMF= natural moisturizing factor. ^1^ parts of the encoding filaggrin gene were not covered and were therefore excluded from analysis. ^2^ The NMF measurement was valid if at least 10 valid NMF values were measured in at least two profiles at different locations.

**Figure S2.** NMF content by *FLG* genotypes


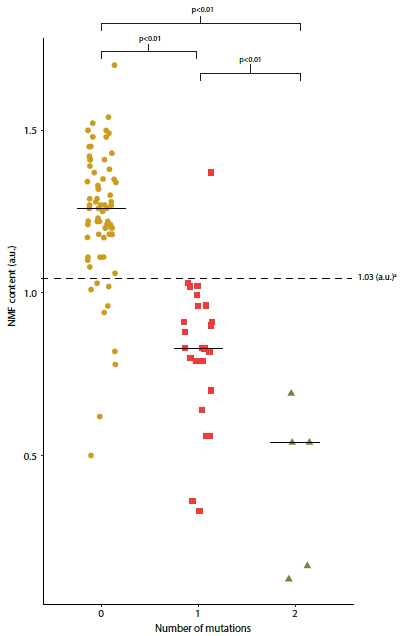


Pairwise comparison showed significant different NMF amount between three *FLG* genotypes (Mann-Whitney U test). Wild-type patients had median NMF content of 1.26 a.u. (IQR 1.18-1.37) and median value of 0.83 (IQR 0.75-0.96) and 0.54 (0.14-0.62), respectively for patients with one and two mutations in *FLG*. ^a^ Cutoff value of 1.03 a.u.
